# Supplementary material for: Association between oral microbiome and seven types of cancers in East Asian population: a two-sample Mendelian randomization analysis
Source: Front Mol Biosci. 2023 Nov 21;10:1327893. doi: 10.3389/fmolb.2023.1327893 (PMC10702768; doi:10.3389/fmolb.2023.1327893)
Supplement: Supplementary file 1 [file DataSheet1.ZIP › Supplementary Materials/MR plots for tongue/tongue═╝/Gastric cancer/pheno.887_to_gastric cancer_forest.pdf]

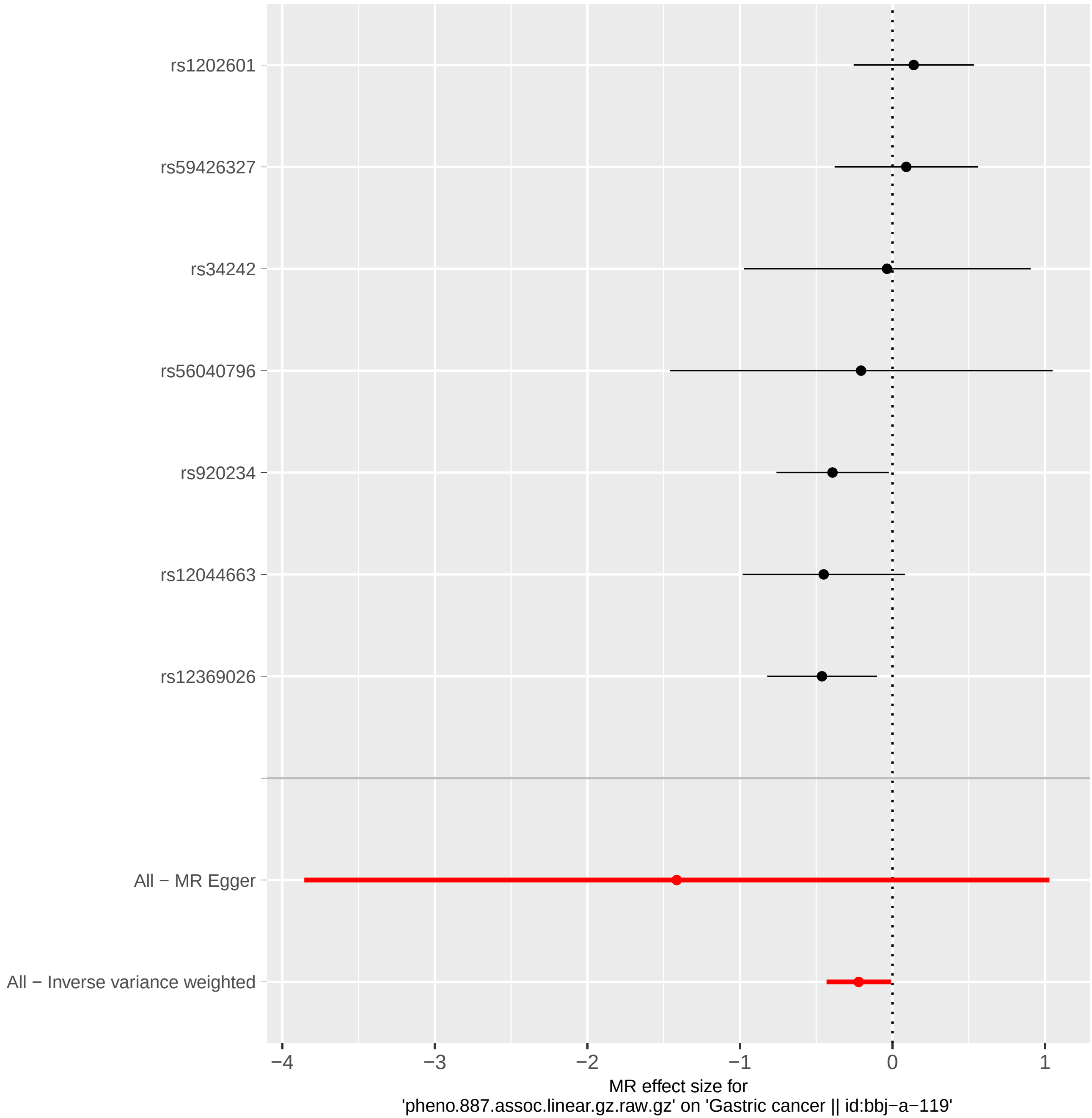
rs1202601
rs59426327
rs34242
rs56040796
rs920234
rs12044663
rs12369026
All − MR Egger
All − Inverse variance weighted
−4
−3
−2
−1
0
1
MR effect size for
'pheno.887.assoc.linear.gz.raw.gz' on 'Gastric cancer || id:bbj−a−119'
